# CLINICAL FEATURES AND PATTERN OF FRACTURES AT THE TIME OF DIAGNOSIS OF OSTEOGENESIS IMPERFECTA IN CHILDREN

**DOI:** 10.1590/1984-0462/;2017;35;2;00001

**Published:** 2017

**Authors:** Evelise Brizola, Marina Bauer Zambrano, Bruna de Souza Pinheiro, Ana Paula Vanz, Têmis Maria Félix

**Affiliations:** a Universidade Federal do Rio Grande do Sul, Porto Alegre, RS, Brasil.; b Hospital de Clínicas de Porto Alegre, Porto Alegre, RS, Brasil.

**Keywords:** osteogenesis imperfecta, bone fracture, clinical features, clinical diagnosis, differential diagnosis

## Abstract

**Objective:**

To characterize the fracture pattern and the clinical history at the time of diagnosis of osteogenesis imperfecta.

**Methods:**

In this retrospective study, all patients with osteogenesis imperfecta, of both genders, aged 0-18 years, who were treated between 2002 and 2014 were included. Medical records were assessed to collect clinical data, including the presence of blue sclerae, dentinogenesis imperfecta, positive familial history of osteogenesis imperfecta, and the site of the fractures. In addition, radiographic findings at the time of the diagnosis were reviewed.

**Results:**

Seventy-six patients (42 females) were included in the study*.* Individuals’ age ranged from 0 to 114 months, with a median (interquartile range) age of 38 (6-96) months. Blue sclerae were present in 93.4% of patients, dentinogenesis imperfecta was observed in 27.6% of patients, and wormian bones in 29.4% of them. The number of fractures at diagnosis ranged from 0 to 17, with a median of 3 (2-8) fractures. Forty (57%) patients had fractures of the upper and lower extremities, and 9 patients also had spinal fractures. The diagnosis was performed at birth in 85.7% of patients with type 3, and 39.3% of those with type 4/5 of the disorder.

**Conclusions:**

Osteogenesis imperfecta is a genetic disorder with distinctive clinical features such as bone fragility, recurrent fractures, blue sclerae, and dentinogenesis imperfecta. It is important to know how to identify these characteristics in order to facilitate the diagnosis, optimize the treatment, and differentiate osteogenesis imperfecta from other disorders that also can lead to fractures.

## RESUMO

**Objetivo:**

Caracterizar o padrão de fraturas e a história clínica no momento do diagnóstico de osteogênese imperfeita.

**Métodos:**

Neste estudo retrospectivo, foram incluídos todos os pacientes com osteogênese imperfeita de ambos os sexos, com idades entre 0 e 18 anos, que realizaram tratamento entre 2002 e 2014. Os prontuários médicos foram revisados para coleta de dados clínicos, incluindo presença de escleras azuladas, dentinogênese imperfeita, história familiar positiva para a doença e locais das fraturas, além de achados radiográficos no momento do diagnóstico.

**Resultados:**

Foram incluídos no estudo 76 pacientes (42 do sexo feminino), com idade, no momento do diagnóstico, entre 0 e 114 meses [mediana (p25-p75) de idade de 38 (6-96) meses]. Escleras azuladas estavam presentes em 93,4% dos pacientes, dentinogênese imperfeita foi observada em 27,6% e ossos wormianos em 29,4%. O número de fraturas ao diagnóstico variou entre 0 e 17, com uma mediana de 3 (2-8) fraturas. Em 40 (57%) pacientes, as fraturas eram de membros superiores e inferiores no momento do diagnóstico e, em 9 pacientes também havia fratura vertebral. O diagnóstico foi realizado ao nascimento em 85,7% dos pacientes com o tipo 3 e em 39,3% daqueles com tipo 4/5 da doença.

**Conclusões:**

Osteogênese imperfeita é uma doença genética com características clínicas distintas, tais como fragilidade óssea, fraturas recorrentes, escleras azuladas e dentinogênese imperfeita. É importante saber identificar essas características, facilitando o diagnóstico, otimizando o tratamento e diferenciando de outras doenças que também podem causar fraturas.

## INTRODUCTION

Osteogenesis imperfecta (OI) is a systemic genetic disorder of connective tissue with prevalence of 6 to 7 per 100,000 births.^1^ OI affects all tissues that contain collagen, mainly bone tissue. Low bone mass is the main characteristic of OI, which causes bones to be brittle and susceptible to deformities and recurrent fractures.^2^ ^,^ ^3^

Most cases of OI are characterized by autosomal dominant inheritance caused by mutations in *COL1A1* or *COL1A2* genes*.* However, recent studies showed that OI can also be caused by mutations in other 19 genes involved in the biosynthesis of collagen or osteoblast function with dominant, recessive, or X-linked inheritance.^1^ ^,^ ^4^

Owing to the extensive genotypic and phenotypic heterogeneity, OI has been classified into several types according to the clinical characteristics, radiological aspects, and the responsible genes.^1^ ^,^ ^3^ The Nosology Group of the International Skeletal Dysplasia Society redefined the traditional clinical classification of OI, adding OI type 5 (OI-5) to the four groups originally described by Sillence.^4^ ^,^ ^5^ ^,^ ^6^

OI type 1 (OI-1) is a mild form, characterized by none or few fractures and minor bone deformities. OI type 2 (OI-2) is the most severe type, characterized by extreme fragility of the bones, leading to death in the neonatal period. OI type 3 (OI-3) is severe; patients have multiple fractures, significant bone deformities, and short stature. OI type 4 (OI-4) is a moderate type with high clinical variability, in which patients can develop few or many fractures associated with bone deformities.^1^ ^,^ ^3^ ^,^ ^5^ People with OI-5 have a moderate form of the disorder with some distinct clinical and radiological features, such as calcification of the interosseous membrane between the radius and ulna, and/or the tibia and fibula, hyperplastic callus formation in long bones, radial head dislocation, and absence of dentinogenesis imperfecta.^1^

Fractures can occur at any stage of life in patients with OI; however, most fractures occur during childhood. Clinical features observed in individuals with OI can also be observed in other genetic and metabolic disorders.^7^ The diagnosis of OI is still made based on clinical and radiological features.^4^ ^,^ ^6^ ^,^ ^7^ Genetic testing is not yet available as routine analysis in many countries and/or there is no insurance or health systems coverage. Consequently, it is important for professionals who provide care for pediatric populations to know how to differentiate and identify these specific cases and be aware of the clinical conditions at the time of diagnosis of this genetic disorder.

This study aimed at characterizing the pattern of fractures and the clinical history at the time of diagnosis of OI in pediatric patients.

## METHOD

In this retrospective study, medical records of male and female patients with OI, who were aged 0-18 years and were being treated at the Reference Center for Osteogenesis Imperfecta at *Hospital de Clínicas de Porto Alegre* (acronym in Portuguese - CROI-HCPA), Porto Alegre, Brazil, between January 2002 and January 2014 were reviewed. Patients with other primary and secondary causes of osteoporosis, such as hypophosphatasia, calcium deficiency, and treatment with glucocorticoids were excluded. This research was approved by the Research Ethics Committee of the *Hospital de Clínicas de Porto Alegre* (number 13-0079), and all patients or guardians signed an informed consent form.

OI diagnosis was based on clinical features and radiological data, according to the criteria established by Sillence.^5^ The data collected included clinical data at the time of diagnosis of OI, such as age, family history of OI, and clinical characteristics of the disorder (blue sclerae, dentinogenesis imperfecta, number and site of fractures). Routine radiographs were reviewed with special attention to the presence of wormian bones in the skull. The reported fractures were classified as present in lower limbs (femur, tibia, or fibula) or upper limbs (radius, ulna, and humerus), and/or other specific site. Our study was limited to the results at the time of diagnosis of OI and did not intend to represent the natural course of the disorder in children.

Continuous variables were expressed as median and interquartile ranges due to the asymmetric distribution of data. Categorical variables were described by absolute and relative frequencies and were compared among the OI type groups, using the chi-square test. The level of significance was set at 5% *(p*≤0.05). Statistical analyses were performed using the software SPSS (version 18.0; SPSS Inc., Chicago, IL, USA).

## RESULTS

**Figure 1:**
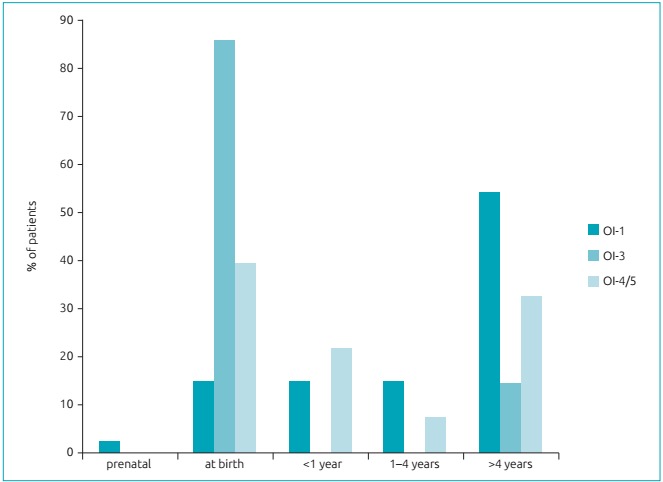
Patient age at the time of diagnosis according to the type of osteogenesis imperfecta.

The sample consisted of 76 patients (42 female) with OI. All cases of OI underwent clinical and radiological diagnosis according to the revised classification of Sillence.^5^ ^,^ ^6^ For statistical analysis, the patient with OI-5 was grouped with those with OI-4, considering that both types represent moderate forms of OI. Table 1 shows the age of patients at the time of diagnosis and clinical features by type of OI. Forty-one (54%) patients were classified as having OI-1, 7 (9%) patients as having OI-3, and 28 (37%) patients as having OI-4/5. The age of the patients at the time of diagnosis ranged from 0 to 114 months, with a median age (p25-p75) of 38 (6-96) months. Most patients (85.7%; n=7) with OI-3, and 39.3% (n=28) of patients with OI-4/5 were diagnosed in the perinatal period; and 68.3% (n=41) subjects with OI-1 were diagnosed between 1 and 5 years of age (Figure 1).

**Table 1:**
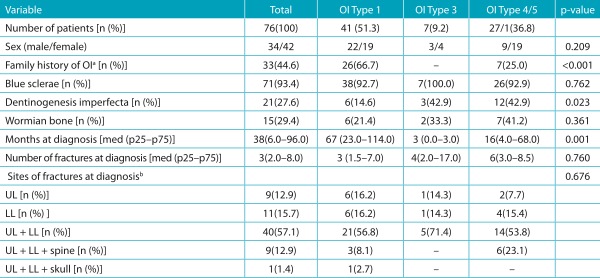
Clinical features at the time of diagnosis by type of osteogenesis imperfecta.

OI: osteogenesis imperfecta; UL: upper limbs; LL: lower limbs; med (p25-p75): median (interquartile range); ^a^unknown family history in two cases; ^b^six children did not present with any fracture at the time of diagnosis.

**Figure 2:**
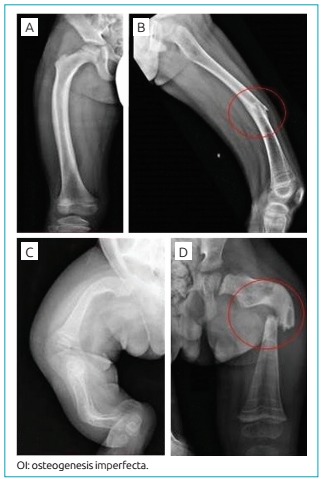
Radiographic findings of the lower limbs. (A and B) Male patient with osteogenesis imperfecta type 1 showing minor bowing of the right femur and diaphyseal fracture of the left femur; (C) female patient with osteogenesis imperfecta type 3 showing severe bowing of the long bones; (D) male patient with osteogenesis imperfecta type 3 showing significant diaphyseal fracture and osteoporosis.

**Figure 3:**
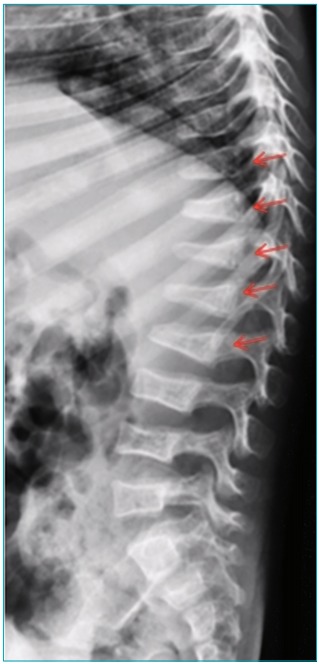
Female patient with osteogenesis imperfecta type 4. Radiographic image of the thoracolumbar spine showing multiple vertebral fractures.

Among the patients, 33 (44.6%) of them had a family history of OI. In two cases, family history was unknown because the children were adopted and there were no data available. Of all patients, 71 (93.4%) had blue sclerae, 21 (27.6%) had dentinogenesis imperfecta, and 15 (29.4%) had wormian bones. The number of fractures at the time of diagnosis ranged from 0 to 17, with a median of 3 (2-8) fractures. Only 6 patients (7.8%) did not have any fracture at the time of diagnosis, and 4 of them had a mild form of OI (OI-1). The majority of patients (n=40; 57.1%) had fractures in the upper and lower limbs (Figure 2); 6 (23.1%) patients with OI-4/5, and 3 patients (8.1%) with OI-1, had suffered additional vertebral fractures (Figure 3). One (1.4%) patient with OI-1 also had a history of skull fracture at the time of diagnosis, caused by an accidental fall from the crib.

## DISCUSSION

Wide phenotypic variability is observed in subjects with OI; however, there is a pattern of fractures and features that helps characterize the disorder clinically and radiologically^8^, and which constitutes the basis for the identification of cases and diagnosis of OI. Genetic testing is useful and provides a better understanding of the disorder; however, it is still not available as a routine test in many treatment centers. As OI is a rare disorder, few professionals have experience in recognizing the specific characteristics of the disorder.

In our sample, the most severe types of OI were diagnosed at an earlier age if compared with the milder types. These results corroborate previous reports, showing that initial fractures tend to occur within the uterus or during the perinatal period; and a high incidence of fractures during growth often causes progressive bone deformity in patients with more severe types of OI.^1^ ^,^ ^3^ Non-lethal forms of OI are often not detected in the prenatal period, and the result of prenatal sonographic diagnosis is not completely reliable.^2^ ^,^ ^9^

Severe and lethal forms of the disorder can be diagnosed by ultrasound during the second trimester of pregnancy, based on the detection of abnormalities in the skull and ribs, micromelia, bowing of long bones, decreased bone echogenicity, fetal growth retardation, ventriculomegaly, polyhydramnios, and even formation of callus, secondary to the occurrence of fracture.^2^ ^,^ ^9^ No patient with OI-2 was diagnosed in our center; however, it is known that approximately 90% of fetuses with OI-2 are stillborn or do not survive the first month after birth, due to the severity of the clinical presentation of the disorder.^1^ ^,^ ^2^

The postnatal diagnosis is based on a family history of OI, clinical and radiological signs, and bone densitometry. Further investigation may include biochemical and molecular analysis.^2^ ^,^ ^10^ Characteristics which are considered classic in OI, such as blue sclerae, wormian bones, dentinogenesis imperfecta, and fractures may be present or not in these patients. In addition, other signs can be observed, such as triangular face, short stature, capsular ligamentous hyperlaxity, cardiovascular and eye abnormalities, hearing loss, platybasia, and basilar invagination.^1^ ^,^ ^2^ ^,^ ^3^ Restriction of the forearm movements, progressive joint contractures, and craniosynostosis can also be observed in OI, which is secondary to rare mutations in the new genes related to OI.^1^ ^,^ ^11^

However, these features are not unique to OI, and may be present in other disorders. Most of our sample (93.4%) had blue sclerae. However, blue sclerae are also reported in healthy children, in individuals with iron deficiency, and in other syndromes, including Loeys-Dietz, De Barsy, Marshall-Smith, Ehler-Danlos, and Russel-Silver.^7^ ^,^ ^12^ ^,^ ^13^ ^,^ ^14^ ^,^ ^15^ ^,^ ^16^ ^,^ ^17^ ^,^ ^18^ ^,^ ^19^ In addition, it is also a clinical sign described in cases of alkaptonuria, brittle cornea syndrome, and in a study on two siblings with VATER association and multiple malformations.^20^ ^,^ ^21^ ^,^ ^22^ ^.^

Wormian bones - small supernumerary bones found between the sutures and fontanelles of the skull - commonly present in OI, can also be observed in the normal pediatric population and other conditions as cleidocranial dysostosis, pycnodysostosis, hypophosphatasia, hydrocephalus, congenital hypothyroidism, and Down syndrome.^12^ ^,^ ^23^ Wormian bones are observed in approximately 30% of children evaluated with OI. Marti and colleagues analyzed brain scans and assessed the frequency of wormian bones in a general pediatric population aged 0-3 years. They observed wormian bones in 53% of children (n=320), and 60 (10%) of them had 4 or more wormian bones.^12^

Dentinogenesis imperfecta was observed in less than a third of the patients in this study. However, many of them were diagnosed with OI before the age at which the primary tooth erupt. Although approximately 50% of children and adults with OI may have dental involvement, dentinogenesis imperfecta is not pathognomonic of this disorder.^24^ ^,^ ^25^Dentinogenesis imperfecta causes structural defects in dentin formation in primary and permanent dentition.^24^ There are three types of dentinogenesis imperfecta, and the type I is associated with OI, being secondary to the defect in type 1 collagen, characterized by typical amber color and translucency of teeth.^24^ ^,^ ^25^

Bone fragility and susceptibility to fractures with no or minimal trauma are typical features of OI. In this study, patients with OI-1 had fewer fractures, and those fractures occurred later, when compared with patients with OI-3 and OI-4/5. It is estimated that 10% of children with OI-1, - the mildest form of the disorder, which is less often associated with deformities of long bones - do not show any fracture during childhood.^2^ ^,^ ^3^ In a previous study, for mild OI cases, we observed that the initial fractures tend to occur during the period in which children begin to walk, because the upright posture promotes increased weight load on the lower limbs, leading to secondary fractures.^26^

In a retrospective study, Greeley and colleagues described the pattern of fractures and clinical features in 68 infants and children under the age of 18 years at the time of diagnosis of OI.^8^ Among the participants, 26 (38%) had no fracture, 22 (32%) had fractures of the extremities, and 15 (22%) had rib fractures. Most of these fractures were diagnosed in the prenatal and perinatal periods. The number of fractures at the time of diagnosis ranged between 1 and 37, and 7 (10%) subjects showed more than 2 fractures. Blue sclerae were observed in 51 (75%) patients, and dentinogenesis imperfecta was observed in only 11 (16%) patients. The authors concluded that the number of fractures, age at the time of diagnosis, and site of fractures are features that may facilitate the diagnosis of OI.^8^

Complete or incomplete fractures of the shaft of long bones and thoracolumbar vertebral compression fractures are observed more frequently in patients with OI.^10^ The prevalence of fractures is high in children with OI; however, the fracture pattern differs from that observed in victims of child physical abuse (CPA).^8^ ^,^ ^10^ Multiple fractures in different stages of consolidation, complex fractures of the skull, sternum, scapula, vertebral spinous processes, and/or posteromedial ribs, as well as intracranial or visceral lesions are lesions described as highly suggestive of CPA.^8^ ^,^ ^10^ CPA cases are much more common than cases of OI. Parents of children with OI often deal with the suspicion of CPA, by health teams during the initial treatment of fractures before the diagnosis is established.^27^ ^,^ ^28^ ^,^ ^29^ ^,^ ^30^However, OI and CPA are not mutually exclusive.^28^

As a differential diagnosis, various conditions that increase the risk of fractures, and disorders that resemble OI with overlap of clinical features, such as metabolic bone disease of prematurity, idiopathic juvenile osteoporosis, Ehlers-Danlos syndrome, hypophosphatasia, idiopathic hyperphosphatasia, osteoporosis-pseudoglioma syndrome, vitamin D and calcium deficiency should be considered.^7^ ^,^ ^8^ In addition, cases of CPA and secondary causes of osteoporosis, including hormone deficiencies, glucocorticoid-induced osteoporosis, and acute lymphoblastic leukemia should be investigated.^7^ ^,^ ^8^

Despite the new findings on the genetic basis of OI, there is no consensus on the use of genetic test as a routine examination for disorder investigation. Zarate et al. suggested, in cases suspected of CPA and OI, to consider genetic testing only in the presence of clinical features of OI, or in cases with a family history of the disorder.^29^ However, in cases of suspected nonaccidental injury, with unexplained fractures in children under investigation for OI, Pepin and Byers suggested evaluating the need for molecular analysis in some cases, considering that the clinical features of the disorder may not be observed during the clinical investigation.^30^

The present study has limitations. It is a retrospective study, based on data recorded in medical records. Some data, particularly the number of fractures at diagnosis, were not documented radiographically, because there was no radiological record available for the occurrence of each fracture, and therefore these data were collected on the basis of clinical histories and medical records. Our results only represent clinical and radiological aspects at the time of diagnosis of OI. They do not represent a pattern of fractures throughout the life of these individuals.

The data presented in this study suggest that fractures associated with OI occur in similar sites, and the number of fractures varies according to the clinical severity of the disorder. Fractures of lower limbs associated with upper limb fractures were more frequent in all types of OI; and the number of fractures was higher in patients with more severe forms of the disorder. The pattern of bone fractures and clinical features are useful information to identify such cases. Health professionals should be aware of the clinical features and the pattern of typical fractures caused by OI, which facilitates the distinction between this disorder and other disorders that lead to bone fragility or cases of CPA.
